# Cancer screening programs in South-east Asia and Western Pacific

**DOI:** 10.1186/s12913-023-10327-8

**Published:** 2024-01-18

**Authors:** Hwee-Lin Wee, Karen Canfell, Han-Mo Chiu, Kui Son Choi, Brian Cox, Nirmala Bhoo-Pathy, Kate T Simms, Chisato Hamashima, Qianyu Shen, Brandon Chua, Niyomsri Siwaporn, Esther Toes-Zoutendijk

**Affiliations:** 1 Saw Swee Hock School of Public Health, National University of Singapore, Singapore, Singapore; 2 The Daffodil Centre, A Joint Venture with Cancer Council NSW and the University of Sydney, Sydney, NSW Australia; 3 Department of Internal Medicine, College of Medicine, National Taiwan University, Taipei, Taiwan; 4 Graduate School of Cancer Science and Policy, National Cancer Center, Ilsandonggu, Goyang Republic of Korea; 5 Dunedin School of Medicine, University of Otago, Dunedin, New Zealand; 6 Centre for Epidemiology and Evidence-Based Practice, Faculty of Medicine, Universiti Malaya, Kuala Lumpur, Malaysia; 7 Division of Cancer Screening Assessment and Management, Institute of Cancer Control, National Cancer Center, Tokyo, Japan; 8 Teikyo University, Tokyo, Japan; 9 Department of Medical Services, Ministry of Public Health, National Cancer Institute of Thailand, Bangkok, Thailand; 10 Population Health Sciences, Bristol Medical School, University of Bristol, Bristol, UK; 11 Department of Public Health, Erasmus MC University Medical Center, P.O. Box 2014, Rotterdam, CA 3000 the Netherlands

**Keywords:** Western pacific, South-East Asia, Cancer screening, Organized screening, Breast cancer, Colorectal cancer, Cervical cancer, Lung cancer, Gastric cancer

## Abstract

**Background:**

The burden of cancer can be altered by screening. The field of cancer screening is constantly evolving; from the initiation of program for new cancer types as well as exploring innovative screening strategies (e.g. new screening tests). The aim of this study was to perform a landscape analysis of existing cancer screening programs in South-East Asia and the Western Pacific.

**Methods:**

We conducted an overview of cancer screening in the region with the goal of summarizing current designs of cancer screening programs. First, a selective narrative literature review was used as an exploration to identify countries with organized screening programs. Second, representatives of each country with an organized program were approached and asked to provide relevant information on the organizations of their national or regional cancer screening program.

**Results:**

There was wide variation in the screening strategies offered in the considered region with only eight programs identified as having an organized design. The majority of these programs did not meet all the essential criteria for being organized screening. The greatest variation was observed in the starting and stopping ages.

**Conclusions:**

Essential criteria of organized screening are missed. Improving organization is crucial to ensure that the beneficial effects of screening are achieved in the long-term. It is strongly recommended to consider a regional cancer screening network.

**Supplementary Information:**

The online version contains supplementary material available at 10.1186/s12913-023-10327-8.

## Introduction

The global burden of cancer is large, with over 19 million cancer diagnoses and almost 10 million cancer deaths in 2020 [1]. Population-level risk of cancer differs due to multiple factors: genetic predisposition, diet, risk behaviour, and exposure to substances, viruses and bacteria [2–6]. Cancer-related burden can be altered through the availability of preventive measures, such as screening and vaccination [6–10]. However, not every cancer type is suitable for screening. Screening for a certain cancer type is recommended only if (1) it leads to benefits (e.g. life-years gained, availability of treatment), (2) these benefits outweigh the associated harms of screening (e.g. overdiagnosis, overtreatment), and (3) has a reasonable ratio between benefits and costs (i.e. cost-effective) [11]. Monitoring and evaluation of cancer screening programs should be conducted to ensure that benefits are achieved and harms are limited, and to improve their efficiency and cost-effectiveness, especially when circumstances change [12].

The World Health Organization (WHO) has published cancer screening guidelines or perspectives on cancer screening, containing screening recommendations, and good practice statements [13–16]. Recommendations have been endorsed for cancer screening in Asia and the Western Pacific, although some are outdated [17–20]. The outcomes of the key performance indicators of programs in the region that were monitored and evaluated differ and are often hard to compare [21–26]. This is likely a result of the different screening program designs and organization of the program in each country or region. An overview of the different cancer screening programs in the region is lacking.

The aim of this study was to perform a landscape analysis of existing cancer screening programs in South-East Asia and Western Pacific.

## Methods

### Regions

Countries belonging to South-East Asia (SEARO) and Western Pacific (WPRO) regions were included in the study, using WHO regional office denominations [27]. All countries and independent regions are listed in Table 1. As a recent review has been published on cancer control in the Pacific Island states, these territories were not part of this study [28].

**Table 1:** Overview of all cancer screening programs in countries, regions and island states in South-East Asia and Western Pacific.*

Country	Design	Cancer types	References
South-East Asia			
Bangladesh	Opportunistic	Cervical, Breast, Oral	[54, 77, 78]
Bhutan	Opportunistic	Cervical	[79, 80]
South Korea	Organized	Breast, Cervical, Colorectal, Gastric, Liver, Lung	[36]
India	Opportunistic	Breast, Cervical, Oral	[81–83]
Indonesia	Opportunistic	Breast, Cervical, Colorectal	[84–86]
Maldives	Opportunistic	Cervical	[87]
Myanmar	Opportunistic	Cervical	[55]
Nepal	Opportunistic	Cervical	[88]
Sri Lanka	Opportunistic	Breast, Cervical, Oral	[89, 90]
Thailand	Organized	Cervical, Colorectal Breast	[33, 37]
Timor-Leste	Unknown		
Western Pacific			
Australia	Organized	Breast, Cervical, Colorectal	[34]
Brunei Darussalam	Organized	Cervical	[91]
	Opportunistic	Breast, Colorectal, Liver, Nasopharyngeal	
Cambodia	Opportunistic	Cervical, Breast	[55, 92]
China	Opportunistic/Organized local initiatives	Breast, Cervical, Colorectal, Gastric, Liver, Lung, Nasopharyngeal, Oesophageal	[23–25, 93–97]
Japan	Organized	Breast, Cervical, Colorectal, Gastric, Lung	[17]
Laos	Opportunistic	Cervical	[55]
Malaysia	Opportunistic	Breast, Cervical, Colorectal	[38, 98, 99]
Mongolia	Opportunistic	Cervical	[100]
New Zealand	Organized	Breast, Cervical, Colorectal	[30]
Papua New Guinea	Unknown		
Philippines	Opportunistic	Breast, Cervical, Colorectal, Prostate	[101]
Singapore	Organized	Breast, Cervical, Colorectal	[32]
Taiwan	Organized	Breast, Cervical, Colorectal, Oral	[22, 31, 102, 103]
Vietnam	Opportunistic	Breast, Cervical, Colorectal, Oral	[104]

* Information presented in Table 1 is derived from the selective narrative review

### Step 1 - literature review

Narrative literature review was used as an exploration to identify countries with organized screening programs. A comprehensive search strategy was carried out by two independent researchers (E.T-Z and B. Chua), and included published literature or national or regional guidelines, all written in English. We searched on PubMed and governmental or cancer society websites. The following search terms were used: “cancer screening Asia”, “cancer screening COUNTRY” or “cancer prevention COUNTRY” or “TYPE of cancer screening COUNTRY”. For each country or region, we searched for information on the design of the program (opportunistic or organized) and the existence of official national or regional cancer screening recommendations or guidelines available in the public domain and/or scientific literature.

If an official screening recommendation was published and available in the public domain, we collected information on the different cancer types and the applied screening strategy (i.e. year of initiation, eligible age, screening modality, and screening interval). Subsequently, we composed a narrative summary of the findings per country or region.

### Expert inputs on organization of the cancer screening programs

Representatives were only contacted if the narrative review showed that their country had an organized program in place. Representatives from countries with only opportunistic cancer screening in place were not contacted. The country or regional representatives were asked to provide relevant information on the organizations of their cancer screening program. These representatives were selected based on review of list of authors in published articles related to cancer screening in the target region. The following topics were addressed: types of cancer screening, initiation of the program, screening test modality, age range and screening interval. Each of the representatives was asked to provide information on the organization of their cancer screening programs by completing the 16 criteria proposed by Zhang et al. [29]. A checklist of the 16 criteria for each of the countries is presented in Supplementary 1.

## Results

### Organization of screening

Most of the countries in South-East Asia and the Western Pacific had a cancer screening program; 15 countries used an opportunistic approach; 8 countries had established an organized program and for 2 countries the program design of cancer screening was unknown or had no cancer screening program. Although most countries had an opportunistic program in place, not all had an official national or regional screening recommendation or guideline in place (Table 1). All countries with an organized program had an existing screening recommendation or guideline on the screening strategy in place. Japan had the longest history of cancer screening programs, starting in 1983 with cervical cancer and gastric cancer screening [17]. Australia, New Zealand and Taiwan have long-standing programs, with breast cancer and cervical cancer screening starting in the late 1980s and early 1990s [21, 22, 30, 31].

For each of the 8 countries that were identified as having an organized approach the level of organization was assessed. Each representative provided information on their screening strategies and completed the checklist of 16 criteria (Table 2 and Supplementary material). All 8 countries had a screening protocol/guideline that describes the target population and screening strategies as well as monitoring and evaluation. All programs also have a policy framework, carry out evaluation of program performance based on indicators, and a system for identifying the target population. Most programs have a system for inviting the eligible target population, with the exception of Malaysia. However, for those that have a system in place, the invitation method varies for each country. For example, Singapore only invites women aged 40 years old for breast cancer screening and not at older ages. Not all countries meet all criteria 8 to 16. Only South Korea, Australia and Taiwan meet all the essential criteria for an organized cancer screening program.

**Table 2:** Checklist of 16 essential criteria for organized cancer screening programs for countries or regions with (partly) organized programs

Country	South Korea	Thailand	Australia	Japan	New Zealand	Singapore	Taiwan	Malaysia
Representative	*Choi*	*Siwaporn*	*Canfell, Simms*	*Hamashima*	*Cox*	*Wee*	*Chiu*	*Bhoo-Pathy*
Essential criteria by Zhang et al. (2022)								
1. Cancer screening program has a protocol/guideline describing at least the target population, screening intervals, screening tests, referral pathway, management of positive cases	Yes	Yes	Yes	Yes	Yes	Yes	Yes	Yes
2. There is a system in place for identifying the target population	Yes	Yes	Yes	Yes	Yes	Yes	Yes	No
3. There is a system in place for inviting eligible individuals for screening	Yes	Yes	Yes	Yes	Yes	No	Yes	No
4. Cancer screening program has a policy framework from the health authorities defining governance structure, financing, goals and objectives of the program	Yes	Yes	Yes	Yes	Yes	Yes	Yes	Yes
5. Performance of screening program should be evaluated with appropriate indicators	Yes	Yes	Yes	Yes	Yes	Yes	Yes	Yes
6. The protocol/guideline should at least describe: monitoring and evaluation	Yes	Yes	Yes	Yes	Yes	Yes	Yes	Yes
7. There is a system in place for notifying the results and informing about follow up	Yes	Yes	Yes	Partially	Yes	Yes	Yes	Yes
8. There is a system in place for sending recall notice to the non-compliant individuals	Yes	No	Yes	Partially	Yes	Partially	Sometimes	No
9. Auditing of the program	Yes	No	Yes	No	No	Yes	Yes	Yes
10. A specified team/organization is responsible for quality assurance/ improvement	Yes	Yes	Yes	No	Yes	Yes	Yes	Yes
11. Performance of cancer screening program is evaluated, published and widely disseminated on a regular basis	Yes	No	Yes	Yes	No	Partially	Yes	Yes
12. All activities along the screening pathway are planned, coordinated and evaluated through a quality improvement framework (quality assurance)	Yes	No	Yes	Yes	No	Yes	Yes	Yes
13. An evidence-based protocol/guideline developed in consensus with majority of stakeholders	Yes	No	Yes	Yes	Yes	Yes	Yes	Yes
14. An information system exists with appropriate linkages (between population databases, screening information, cancer registry, etc.) for screening implementation and evaluation	Yes	No	Yes	No	Yes	No	Yes	No
15. The screening program has a provision of continued training for service providers	Yes	No	Yes	Partially	No	Yes	Yes	Yes
16. Performance of screening program should be evaluated with reference standards for the indicators	Yes	No	Yes	Yes	No	Yes	Yes	No

### Cancers

An overview of the organized programs is presented below, stratified by cancer type (cervical cancer, breast cancer, colorectal cancer, gastric cancer, and cancers in high-risk groups).

#### Cervical cancer screening

**Fig. 1:**
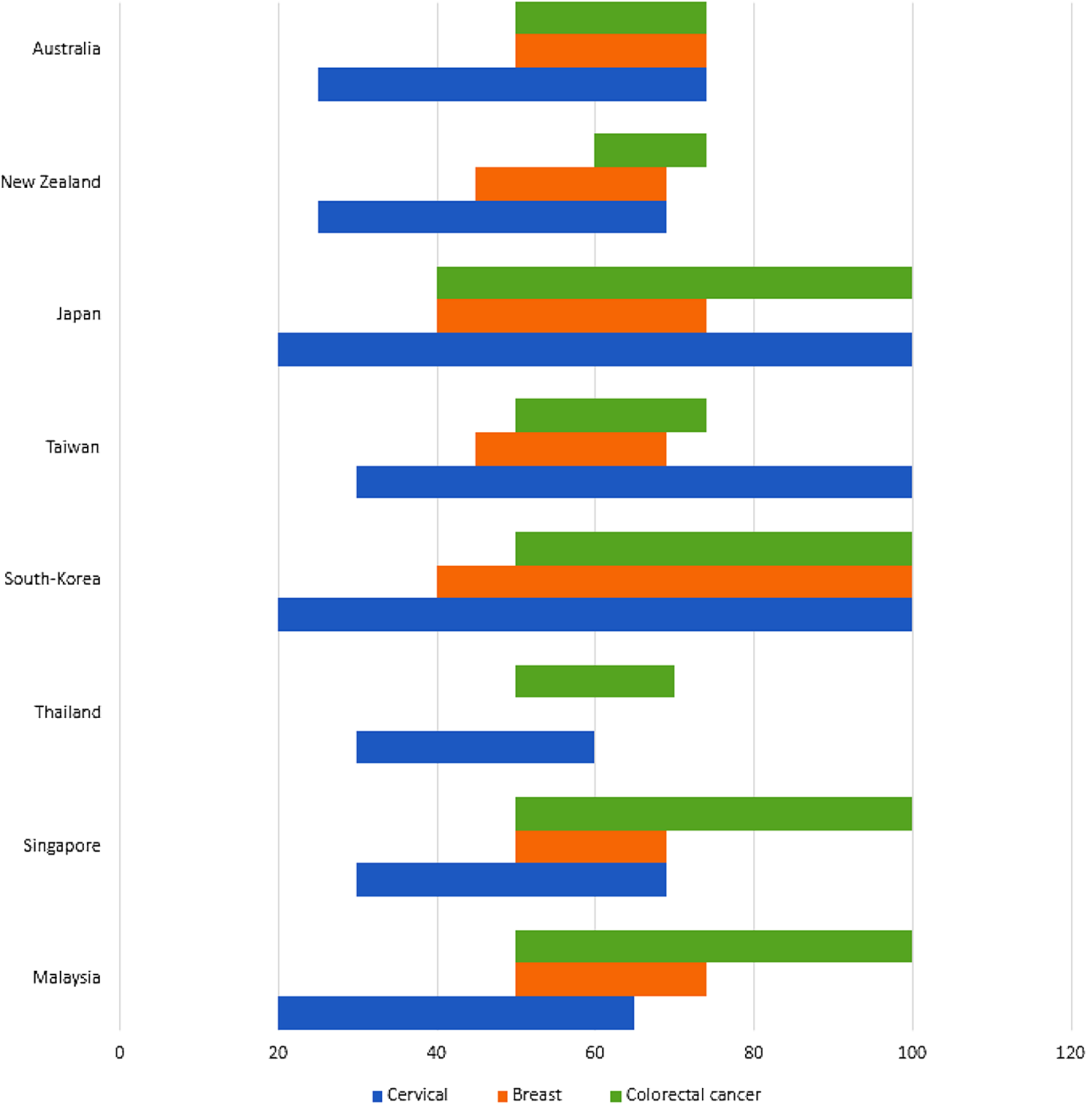
Overview of age range in organized breast, cervical and colorectal cancer screening programs

Remarkably, all countries listed in Table 1 had a cervical cancer screening strategy in place, either Papanicolaou test (Pap Smear), Visual Inspection with Acetic Acid (VIA) test or Human Papillomavirus (HPV) testing, except for the two countries (Timor-Leste and Papua New Guinea) for which no data on cancer screening was available in the literature. For the organized cervical cancer screening programs, the screening strategy differed among the countries. Most countries used the Pap smear as the preferred screening modality. Thailand, Australia and Singapore recently switched from Pap Smear to HPV testing [32–34]. In Thailand HPV screening was introduced in 2020, but has not been fully implemented across the whole country and cytology screening is still the main screening modality in large part of the country (*personal communication*). New-Zealand switched to HPV testing in September 2023. Japan has evaluated the current cytology testing strategy and plans to implement HPV testing. Implementation is halted by the government and is not officially been recommended as screening modality. When only considering the countries with an organized program, the start and stop age of screening differed among the countries, partly contributable to the chosen screening test (PAP smear versus HPV testing). For the organized programs using Pap Smear, the starting age varied between 20 and 30 years of age (Fig. 1; Table 3). Japan and South Korea had the lowest starting age of 20 years [17, 35]. The stopping age varied between 65 and 69 years of age. For the organized programs using HPV testing, starting age varied between 25 and 30 years and stopping age varied between 60 and 74 years, with Australia having the highest stopping age of 74 years [34]. Both Japan and South Korea had no stopping age in their screening guidelines [17, 36]. The recommended screening interval also differed for the test used within the program. For programs using Pap Smear, triennial interval was used except for Japan and South Korea with a biennial interval (Table 2). For all countries with an organized program using HPV testing, the interval was 5 years.

**Table 3:** Overview of cancer screening strategies for countries or regions with (partly) organized programs*

Country	Initiation	Cancer types	Test modality	Age range	Interval	Additional information
South Korea	2002	Breast	Mammography	40 and above	2 years	
	2002	Cervical	Pap Smear	20 and above	2 years	
	2003	Liver	Abdominal Ultrasonography + Serum Alpha-Fetoprotein test	40 and above (high-risk individuals)	6 months	High risk is considered HBsAg positive or anti-HCV positive or liver cirrhosis
	2004	Colorectal	FIT	50 and above	1 year	
	2002	Gastric	UGIS/GE	40 and above	2 years	
	2019	Lung	Low dose CT scan	54–74 with 30 pack-years	2 years	
Thailand#	2005	Cervical	HPV (since 2020)	30–60	5 years	HPV-testing is not fully implemented across the country
	2018	Colorectal	FIT (20 µg Hb/g feces)	50–70	2 years	
Australia	1991	Breast	Mammography	50–74	2 years	
	1991	Cervical	HPV	25–74	5 years	
	2006	Colorectal	FIT (20 µg Hb/g feces)	50–74	2 years	
Japan$	1987	Breast	Mammography	40 and above	2 years	
	1983	Cervical	Pap Smear	20 and above	2 years	HPV-testing is currently debated, but not yet implemented.
	1992	Colorectal	FIT (cut-off not defined)	40 and above	1 year	
	1983	Gastric	UGIS/GE	UGIS: 40 and above GE: 50 and above	UGIS 1 year GE 2 year	
New Zealand	1989	Breast	Mammography	45–69	2 years	
	1991	Cervical	Pap Smear	25–69	3 years	Switching to 5-yearly HPV-testing in 2023
	2017	Colorectal	FIT (40 µg Hb/g feces)	60–74	2 years	
Singapore	2002	Breast	Mammography	50–69	2 years	
	2004	Cervical	Pap Smear HPV	25-29 30-69	3 years 5 years	
	2011	Colorectal	FIT (10 µg Hb/g feces) or colonoscopy	50 and above	1 year 10 years	
Taiwan	2004	Breast	Mammography	45–69 (40–44 with family history)	2 years	
	1995	Cervical	Pap Smear	30 and above	3 years	
	2004	Colorectal	FIT (20 µg Hb/g feces)	50–74	2 years	
	2004	Oral	Oral mucosa inspection	30 and above with the habits of smoking and/or betel nut chewing 18 and above for aborigines with the habit of betel nut chewing	2 years	
*Opportunistic approach with national screening recommendation with work in progress to implement organized cancer screening*						
Malaysia	2011	Breast	CBE/Mammography	35 and above 50–74	1 year 2 years	
	1995	Cervical	Pap Smear	20–65	3 years	HPV-testing was introduced in 2020, but not available yet in the whole country
	2014	Colorectal	FIT	50 and above	1 year	

Abbreviations: Pap Smear (Papanicolaou test); HPV (human papillomavirus); FIT (fecal immunochemical testing); CBE (clinical breast examination); UGIS (Upper Gastro-Intestinography series); GE (Gastrointestinal Endoscopy)

* The information in Table 2 has been provided by the representatives of each country. This information may differ from the references listed in Table 1, which may be out of date

# Thailand has no organized breast cancer screening program

$ Japan has no organized lung cancer screening program

#### Breast cancer screening

Breast cancer screening is not widely adopted in the South-East Asian and Western Pacific region. In all countries with an organized program, mammography was the recommended test modality. There was one exception, Thailand, where clinical breast examination (CBE) was used as the primary screening modality in an opportunistic screening approach, due to limited healthcare capacity and infrastructure [37]. Starting age varied between 40 and 50 years of age (Fig. 1). Stopping age varied between 69 and 74 years of age. Like cervical cancer screening, few countries had no upper age limit. All mammography-based programs used a two-year screening interval.

#### Colorectal cancer screening

All eight organized programs recommended colorectal cancer (CRC) screening, using FIT as the primary test modality. Singapore offered a choice, with colonoscopy as alternative screening modality [32]. Considering FIT only, Japan had the lowest starting age, recommending screening at 40 years of age [17]. All other countries recommended to start at the age of 50, except for New Zealand that recommends starting at the age of 60 (Fig. 1) [21, 30, 32–34, 36, 38]. New Zealand and Australia had the highest stopping age, recommending stopping screening at the age of 74. South Korea, Japan and Singapore had no recommended stopping age for CRC screening. All FIT-based programs used different cut-offs for a positive test, ranging from 10 to 40 µg Hb/g feces, with a one- or two-year screening interval. In Japan and South-Korea, FIT cut-off was not defined on a national level, but differ between regional screening organizations or laboratories. The countries that offered colonoscopy as an alternative screening modality used a 10-year screening interval.

#### Gastric cancer screening

Two countries offered gastric cancer screening to the general population. South Korea and Japan recommend either upper gastro-intestinography series (UGIS) or a gastrointestinal endoscopy (GE) [17, 36]. Both countries offer gastric cancer screening from the age of 50. Both have no stopping age and use a screening interval of two years.

#### Cancer screening in high-risk groups

Cervical, breast, colorectal, and gastric cancer screening are all offered to the general population. Some countries also offered organized programs for high-risk groups. In Taiwan, oral cancer screening is offered biennially to betel nut chewers of 30 years and above [39]. In South Korea, lung cancer screening is offered to smokers with 30 pack years, offering biennially low-dose CT scan to individuals aged 54 to 74 years [40]. In South Korea, liver cancer screening is offered to high-risk individuals aged 40 years and older using an abdominal ultrasonography plus serum alpha-fetoprotein test. High-risk individuals are defined as hepatitis B (HBsAg positive), hepatitis C (anti-HCV positive) or liver cirrhosis patients [41]. Australia plans to introduce lung cancer screening by 2025. In Taiwan, lung cancer screening is still in the pilot phase and has not yet been rolled out to the entire eligible population. Japan only offers lung and prostate cancer screening in an opportunistic manner.

## Discussion

This study presents an overview of cancer screening programs in the South-East Asia and the Western Pacific. Great variations in the offered screening strategies in the region were observed, in which several factors seem to play a part. Most strategies reflect the disease incidence and availability of resources. Many programs lack structured organization, using an opportunistic screening approach. Among the organized screening programs, there is variety in start and stopping age (or even lack of stopping age); which is only partly explained by the choice of screening test.

Using the criteria checklist for organized screening from Zhang et al., we showed that only South Korea, Australia and Taiwan met all the 16 criteria. Still, most of the countries have guidelines, protocol and frameworks in place for a well-organized program. Upfront, although everything appears to be well-regulated in theory, some essential program aspects are missing.

Most cancer types that were screened for, i.e. cervical cancer, breast cancer and colorectal cancer, are like recommended programs in Europe [42]. The most obvious exception was screening for gastric cancer, present in South Korea and Japan. The main reason might be the higher gastric cancer incidence in East-Asia, due to the presence of risk factors [43, 44]. The decision to implement gastric cancer screening is also related to factors other than the incidence: invasiveness of the test, availability of the required endoscopy resources and effectiveness of alternative screening modalities [45]. It has been suggested that testing for the presence of a Helicobacter pylori infection can be used as risk stratification. Combined with endoscopic screening, individuals with high or low risk for gastric cancer can be identified, offering high-risk individuals more intensified screening than low-risk individuals [46].

Screening for oral cancer in Taiwan is another example of a different screening strategy compared to other parts of the world. This example of screening of only high-risk individuals (i.e. having the habit of betel nut chewing or aboriginal people) may serve as an example for future risk-based cancer screening strategies. Currently, most countries offer uniform screening to the target population, but it is expected that it might shift to more personalized screening, focusing on those at highest risk [47–50]. Learning from this screening program for high-risk individuals may be informative for many other countries.

In most countries, the age range and frequencies for specific cancer screening recommendations are related to the screening test used; for instance, intervals are longer in countries that use primary HPV compared to those that use pap smear [13, 51]. In Japan and South Korea, however, cervical cancer screening is recommended at the age of 20 years without upper age limit. The decision on when to stop screening in elderly is controversial, not least when a program has limited resources, also in countries with high resources. The controversial issue is balancing the benefits against the harms, especially at older ages. The benefits of screening, such as mortality reduction, could be smaller at older age due to shorter life expectancy, whereas harms of screening (i.e. complications, additonal testing and associated costs) might be more impactful. All these factors should be weighed properly, when determining the age to stop screening [52]. To be able to measure the benefits and harms of screening, regular program monitoring and evaluation of the key program outcomes, including cost-effectiveness analysis, is crucial to determine the optimal screening strategy for each individual country.

This study revealed that offering well-organized cancer screening in low- and middle-income countries (LMICs) remains a challenge. The challenges of cancer screening in LMICs have been extensively described in the literature [53–55]. Because of limited health system resources and competing health risks, a trade-off on the types of cancer screening to be offered may be necessary [54]. Thailand, for instance, offers the latest cervical cancer screening test (i.e. HPV testing), but does not offer breast cancer screening (i.e. mammography) due to various reasons. This also reflects the choice for the best screening modality, of which mammography might not be considered as first choice. Recently it has been shown that CBE can be an effective screening tool, however, the target population should be carefully chosen (i.e. women aged > 50 years) [56]. Besides the choice for the cancer type as well as considering alternative screening tests, other preventive measures should be considered in LMIC. For cervical cancer screening specifically, governments should consider the potential of other preventive measures such as HPV vaccination [57].

The narrative review also revealed that standardized evaluation of the program performance is lacking in the region [58–60]. The assumption that an organized program design could facilitate program evaluation does not hold for all countries. For instance, in Singapore, a well-organized and wealthy country, there is no information system that linkages population databases, screening information, and cancer registry data for screening evaluation. To allow for a program comparison, uniform definition of key performance indicators is essential, as can be learned from the cancer screening report in the European Union [42]. Similar accounts for cost-effectiveness analyses. Although we identified a fair amount of literature on CEA of cancer screening programs during the narrative review, only few CEAs have been used for policy decision making [61–73]. A good example of evidence on cost-effectiveness of organized cancer screening has been shown by Lew et al. (2019), providing an overview of modelling estimates of their organized breast, cervical and colorectal cancer screening program in Australia [74]. Reporting standardized screening outcomes and using CEAs to inform policy makers are important topics for future research. All the above underlines the relevance for collaboration between cancer screening researchers in the region.

This study had some limitations that should be addressed. Firstly, we might have overlooked national or regional cancer screening guidelines because we did not include non-English documentations. Therefore, this narrative review may not be fully comprehensive, especially as no formal systematic review was conducted. Secondly, there might be a delay in the publication of new or recently revised guidelines, implying that recommended screening strategies can be outdated. We addressed both limitations by approaching many representatives of countries with (partly) organized programs requesting or verifying information on their national or regional cancer screening programs. This showed to be relevant, as it turned out that what appeared to be an organized program in publications was only using an opportunistic approach, i.e. Malaysia.

Some countries indicated that they have an organized screening program in their countries, but we have suggested otherwise [75, 76]. Readers or policy makers in those countries may disagree with our assessment. This emphasizes the need for a standardized assessment of the organizational structure of different cancer screening programs, as was done for CanScreen5, which provides a comprehensive overview of cancer screening programs worldwide.

In conclusion, this overview showed that there is large variation in cancer screening strategies in South-East Asia and the Western Pacific, with only a few fully organized cancer screening programs. Most cancer screening programs offered screening for common cancer types (i.e. cervical, breast and colorectal cancer). Screening strategies differed, often related to the choice of screening test, although some countries had more intensive screening strategies than other countries. We stronly recommend the establishment of a regional cancer screening network, in which knowledge and experience can be exchanged.

### Electronic supplementary material

Below is the link to the electronic supplementary material.

Supplementary Material 1

Supplementary Material 2

Supplementary Material 3

Supplementary Material 4

Supplementary Material 5

Supplementary Material 6

Supplementary Material 7

Supplementary Material 8

## Data Availability

Data and materials that support the findings of this study are available from the corresponding author (ETZ), upon reasonable request.
